# Subverting Toll-Like Receptor Signaling by Bacterial Pathogens

**DOI:** 10.3389/fimmu.2015.00607

**Published:** 2015-12-01

**Authors:** Victoria A. McGuire, J. Simon C. Arthur

**Affiliations:** ^1^Division of Cell Signalling and Immunology, School of Life Sciences, University of Dundee, Dundee, UK

**Keywords:** MAPK, NF-κB, bacterial effector, signaling, TLR, bacterial pathogen, virulence

## Abstract

Pathogenic bacteria are detected by pattern-recognition receptors (PRRs) expressed on innate immune cells, which activate intracellular signal transduction pathways to elicit an immune response. Toll-like receptors are, perhaps, the most studied of the PRRs and can activate the mitogen-activated protein kinase (MAPK) and Nuclear Factor-κB (NF-κB) pathways. These pathways are critical for mounting an effective immune response. In order to evade detection and promote virulence, many pathogens subvert the host immune response by targeting components of these signal transduction pathways. This mini-review highlights the diverse mechanisms that bacterial pathogens have evolved to manipulate the innate immune response, with a particular focus on those that target MAPK and NF-κB signaling pathways. Understanding the elaborate strategies that pathogens employ to subvert the immune response not only highlights the importance of these proteins in mounting effective immune responses, but may also identify novel approaches for treatment or prevention of infection.

## Introduction

Innate immunity provides the first line of defense against invading pathogens. Recognition of microbial ligands, or pathogen-associated molecular patterns (PAMPs) by pattern-recognition receptors (PRRs), stimulates innate immune cells to upregulate the expression of cytokines, chemokines, and proteins that directly target microbes. Toll-like receptors (TLRs) have been well studied amongst the PRRs, with 10 described in human and 12 in mouse (1). TLRs on the cell surface recognize ligands from extracellular microbes, such as peptidoglycan by TLR1/TLR2, lipoprotein by TLR2/6, lipopolysaccharide (LPS) by TLR4, and flagellin by TLR5. TLR3, TLR7, TLR8, and TLR9 are located in intracellular vesicles where they recognize microbial nucleic acids.

**Figure 1:**
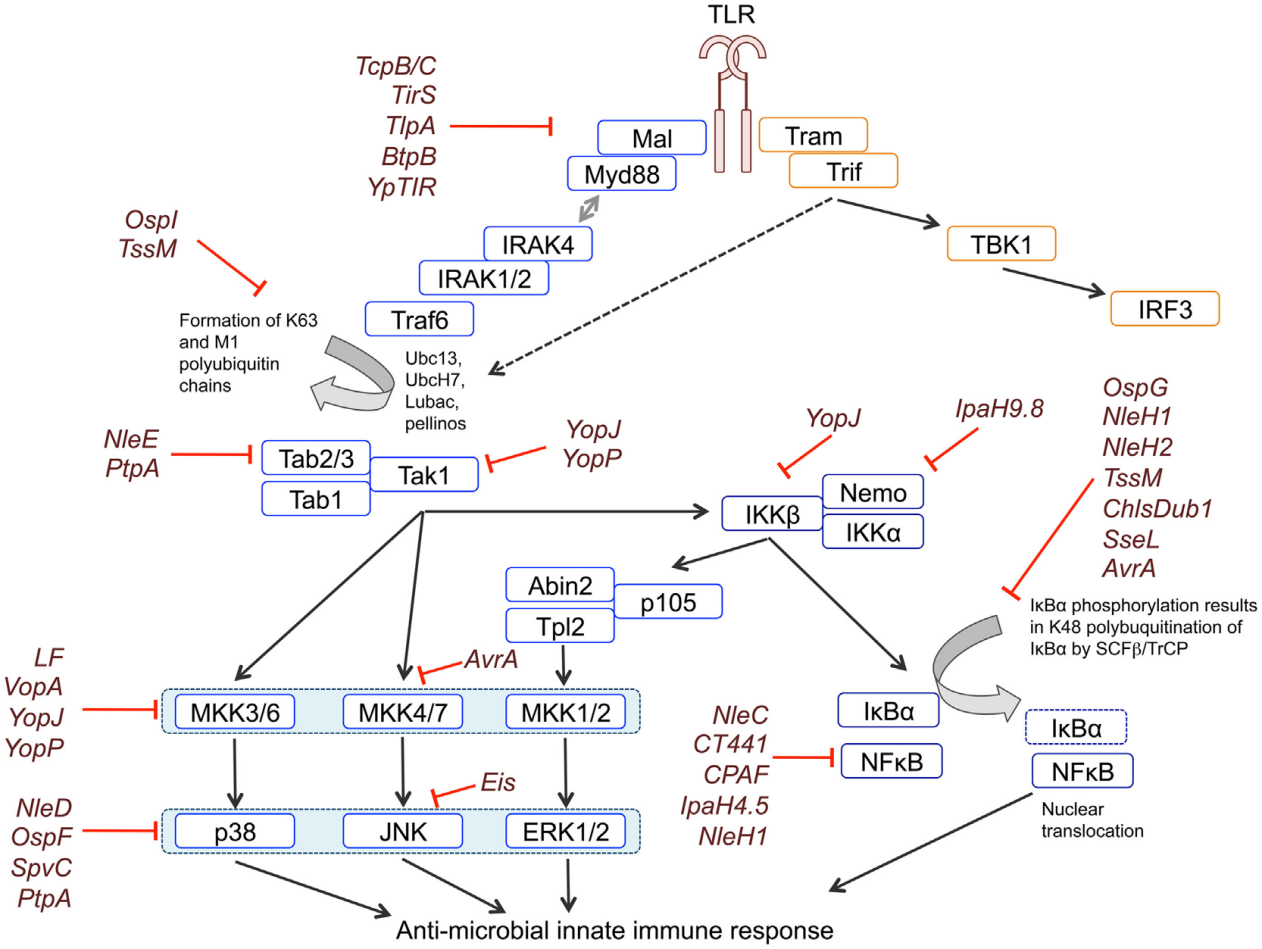
**Blockade of MAPK and NFκB signaling by bacterial effectors**. TLR signaling is initiated by the recruitment of adaptor proteins to the TIR domain of the receptor. Recruitment of MyD88 facilitates Myddosome formation through binding of IRAK4, IRAK1, and IRAK2. IRAKs bind to and recruit the E3 ubiquitin ligase TRAF6, which – perhaps with input from other E3s – generates lysine-63 (K63) linked polyubiquitin chains. K63 linked polyubiquitin chains are used as a substrate by LUBAC to form M1-K63 hybrid polyubiquitin chains. K63 and M1-K63 polyubiquitin chains are necessary for downstream signaling mediated by TAK1. TAK1 phosphorylates and activates IKKα/β, which form the IKK complex together with NEMO/IKKγ. The IKK complex phosphorylates IκBα, resulting in its K48-linked polyubiquitination and proteasomal degradation, which releases the p65 NFκB subunit from inhibition. The IKK complex also phosphorylates p105, generating the p50 NFκB subunit, and enabling the active p50-p65 NFκB dimer to translocate to the nucleus. TAK1 also controls activation of the ERK1/2, p38, and JNK MAPK pathways by acting as a MAP3K for the p38 and JNK pathways and controlling the activation of ERK1/2 via Tpl2. Phosphorylation of p105 by the IKK complex releases Tpl2 from inhibition, allowing Tpl2 to activate ERK1/2 signaling. MAPKs phosphorylate their own downstream targets including other kinases and transcription factors that regulate transcription. Activation of TLR3 and TLR4 can also recruit the TRIF adaptor, which activates NFκB and MAPK signaling via both Receptor Interacting Protein 1 (RIP1) and TRAF6 upstream of TAK1, and activates IRF3 via IKKϵ and Tank-binding kinase 1 (TBK1). Bacterial effectors block signaling by interfering with different components of the signaling cascades, as indicated in the figure.

Stimulation of all TLRs activates the mitogen-activated protein kinase (MAPK) and Nuclear Factor-κB (NF-κB) signaling pathways, both of which are critical for an effective immune response. The current understanding of the signaling events that trigger MAPK and NF-κB activation in response to TLR stimulation have been reviewed recently (1–4), but is summarized below and in Figure 1.

Following detection of PAMPs by a TLR, signaling is initiated by the recruitment of adaptor proteins to the cytoplasmic Toll and IL-1 Receptor (TIR) domain of the receptor. Two main pathways of TLR signaling exist, defined on their use of either the MyD88 (myeloid differentiation primary-response protein 88) or TRIF (TIR domain-containing adaptor protein inducing interferon α/β) adaptor, with all TLRs except TLR3 able to utilize the MyD88 pathway. MyD88 recruits IL-1 receptor-associated kinase (IRAK) 4, IRAK1 and IRAK2 to form a complex known as the Myddosome, which subsequently recruits the E3 ubiquitin ligase TNF receptor-associated factor 6 (TRAF6).

TRAF6 and/or other E3 ubiquitin ligases generate lysine-63 (K63) linked polyubiquitin chains that are used by the linear ubiquitin chain assembly complex (LUBAC) to generate linear (M1)-K63 hybrid polyubiquitin chains. The formation of both K63 and M1-K63 hybrid polyubiquitin chains is required to assemble the signaling complexes that activate downstream pathways. TAK1 plays a central role in activating downstream signaling pathways. First, it phosphorylates and activates IκB kinases (IKKα/β), which form the IKK complex together with NEMO/IKKγ. The IKK complex phosphorylates IκBα, enabling its recognition by the E3 ligase complex SCF-βTrCP (SKP1–cullin-1–F-box complex containing βTrCP), resulting in its K48-linked polyubiquitination and proteasomal degradation. Loss of IκBα releases the p65 NFκB subunit allowing it to translocate to the nucleus.

TAK1 also controls activation of the ERK1/2, p38, and JNK MAPK pathways. MAPK activation requires a cascade of at least three kinases. MAPKs are activated by a MAPK Kinase (MAP2K), which itself is activated by phosphorylation by an upstream MAPK Kinase Kinase (MAP3K). TAK1 acts as a MAP3K for the p38 and JNK pathways and via IKK it controls the activation of Tpl2, the MAP3K that activates ERK1/2 downstream of TLRs. Tpl2 activity is controlled by p105, which tethers it in an inactive complex with Abin2. Phosphorylation of p105 by the IKK complex, releases this complex, allowing Tpl2 to activate its substrates. MAPKs phosphorylate their own downstream targets including other kinases and transcription factors that regulate transcription (1–4).

Pathogenic bacteria have evolved elaborate strategies to perturb intracellular signaling pathways that activate the host immune response. This review describes the mechanisms bacteria use to inhibit TLR-dependent signaling, focusing on strategies that block MAPK and NF-κB signaling and is summarised in Table 1. Interestingly, some intracellular bacteria can also activate MAPK or NF-κB pathways to their advantage at different stages of infection. For example, while within cellular vacuoles, *Salmonella* Typhimurium expresses the kinase SteC which phosphorylates MKK1/2 on Ser200 in the kinase domain (5). Phosphorylation of Ser200 causes MKK1/2 to autophosphorylate on Ser218 and Ser220, leading to activation of ERK1/2 and resulting in reorganization of the actin cytoskeleton, which restrains bacterial growth to control bacterial virulence (5). *S*. Typhimurium also uses SopE, SopE2, and SopB, which act redundantly to activate MAPK and NF-κB via Rho-family GTPases and stimulate inflammation (6). Infection of alveolar macrophages with *Legionalla pneumophila* causes Legionnaire’s disease. *L. pneumophila* translocates the kinase LegK1 into macrophages where it activates NF-κB signaling to inhibit apoptosis and promote intracellular bacterial replication (7). LegK1 phosphorylates a number of proteins in both the canonical and non-canonical NF-κB pathway, including IκBα, IκBβ, IκBϵ, p100 (NFBK2), and p105 (NFKB1) (7). Phosphorylation of IκBα on serines 32 and 36 stimulate its degradation and promote translocation of NF-κB to the nucleus, while phosphorylation of p100 on serines 866 and 870 causes its cleavage to generate the p52 subunit and induce formation of the p52/RelB non-canonical NF-κB complex.

**Table 1:** **Mechanisms used by bacteria to inhibit TLR-dependent signaling by blocking MAPKs or NFκB**.

Protein function	Protein	Bacterial species	Disease	Mechanism	Reference
TIR mimic	TlpA	*Salmonella* Enteritidis	Gastrointestinal disease	Postulated to compete with endogenous TIR domains to prevent signaling	Newman et al. (8)
	TirS	*Staphylococcus aureus*	Skin, respiratory tract, and GI tract infections	Blocks TLR2 signaling	Askarian et al. (9)
	TcpC	*Escherichia coli* CFT073 (UPEC)	Urinary tract infection	Binds MyD88 to prevent downstream signaling	Cirl et al. (10)
	TcpB/BtpA	*Brucella melitensis*	Brucellosis	Mimics Mal (TIRAP) to block TLR2/TLR4 signaling; targets Mal for proteasomal degradation	Cirl et al. (10), Radhakrishnan et al. (13), Sengupta et al. (14)
	BtpB	*Brucella melitensis*	Brucellosis	Interacts with MyD88 to block TLR signaling	Salcedo et al. (12)
	YpTIR	*Yersinia pestis*	Plague	Interacts with MyD88 to block TLR signaling	Rana et al. (11), Spear et al. (66)
Protease	LF	*Bacillus anthracis*	Anthrax	Cleaves MKKs within MAPK-docking domain	Duesbery et al. (15), Vitale et al. (16)
	NleD	*Escherichia coli* (EPEC/EHEC)	Gastrointestinal disease	Cleaves JNK and p38 within TxY dual phosphorylation motif	Baruch et al. (17)
	NleC	*Escherichia coli* (EPEC/EHEC)	Gastrointestinal disease	Cleaves amino-terminus of p65 NF-κB targeting it for proteasomal degradation	Yen and Ooka (18), Mühlen et al. (19), Baruch et al. (17), Pearson et al. (20)
	CT441	*Chlamydia* spp.	Urogenital tract infection, trachoma eye disease	Cleaves p65 NF-κB	Lad and Yang (21)
	CPAF	*Chlamydia* spp.	Urogenital tract infection, trachoma eye disease	Cleaves p65 NF-κB	Christian et al. (23)
Acetyltransferase	VopA	*Vibrio parahaemolyticus*	Gastrointestinal disease	*O*-acetylates MKKs in the activation loop to compete with phosphorylation; *N*-acetylates MKKs in the catalytic loop to disrupt ATP binding	Trosky et al. (24, 25)
	AvrA	*Salmonella* Typhimurium	Gastrointestinal disease	*O*-acetylates MKKs in the activation loop to compete with phosphorylation	Jones et al. (26)
	YopJ/YopP	*Yersinia* spp.	Plague/Yersiniosis	*O*-acetylates MKKs, TAK1 and IKKα and IKKβ in the activation loop to compete with phosphorylation	Orth et al. (28), Mittal et al. (30), Mukherjee et al. (29), Haase and Richter (32), Thiefes et al. (33), Paquette and Conlon (31), Meinzer et al. (34)
	Eis	*Mycobacterium tuberculosis*	Tuberculosis	*N*-acetylates DUSP16/MKP7 to block JNK activation	Kim et al. (35)
Phosphothreonine lyase	OspF	*Shigella* spp.	Dysentery	Removes phosphothreonine in the TxY activation loop of MAPKs	Li et al. (37)
	SpvC	*Salmonella* Typhimurium	Gastrointestinal disease	Removes phosphothreonine in the TxY activation loop of MAPKs	Mazurkiewicz et al. (38)
Kinase/phosphatase	OspG	*Shigella*	Dysentery	Binds to ubiquitin and E2-ubiquitin conjugates; prevents IκBα degradation	Kim et al. (43), Zhou et al. (44)
	NleH1	*Escherichia coli* (EPEC/EHEC)	Gastrointestinal disease	Inhibits IκBα degradation; binds to RPS3 to antagonize NF-κB activity	Gao and Wan (47), Royan et al. (46)
	NleH2	*Escherichia coli* (EPEC/EHEC)	Gastrointestinal disease	Inhibits IκBα degradation	Royan et al. (46)
	PtpA	*Mycobacterium tuberculosis*	Tuberculosis	Dephosphorylates p38 and JNK; competes with ubiquitin for TAB3 binding	Wang et al. (61)
E3 ligase	IpaH9.8	*Shigella*	Dysentery	Targets NEMO and MAPKK (Ste7) for degradation	Rohde et al. (48), Ashida et al. (50)
	IpaH4.5	*Shigella*	Dysentery	Targets NF-κB p65 for ubiquitination, preventing transcription	Wang et al. (51)
	IpaH0722	*Shigella*	Dysentery	Targets TRAF2 for ubiquitination, preventing PKC-induced NF-κB activity	Ashida et al. (52)
Deubiquitylase	SseL	*Salmonella* Typhimurium	Gastrointestinal disease	Prevents Lys48-linked ubiquitination and degradation of IκBα	Le Negrate et al. (57)
	ChlsDub1	*Chlamydia trachomatis*	Trachoma eye disease	Prevents Lys48-linked ubiquitination and degradation of IκBα	Le Negrate et al. (57)
	TssM	*Burkholderia pseudomallei*	Melioidosis	Prevents Lys63-linked ubiquitination of TRAF6/TRAF3 and Lys48-linked ubiquitination and degradation of IκBα	Shanks et al. (58) Tan et al. (60)
Glutamine deamidase	OspI	*Shigella flexneri*	Dysentery	Deamidates glutamine residue in Ubc13 to prevent TRAF6 binding	Sanada et al. (62)
Cysteine methyltransferase	NleE	*Escherichia coli* (EPEC)	Gastrointestinal disease	Targets Npl4 zinc finger domains of TAB2/3 to prevent binding to Lys63-linked polyubiquitin and TAK1 activity	Zhang et al. (63)

## Blocking Signaling by Mimicking TIR:TIR Interactions

A number of bacteria target the initial stage of TLR activation by expressing TIR-containing proteins (Tcps) that interfere with TIR–TIR interactions. A bioinformatics screen for bacterial proteins with homology to human TIRs identified the first TIR-containing protein as TIR-like protein A (TlpA) from *Salmonella enterica* serovar Enteritidis (*Salmonella* Enteritidis), which causes food-borne gastroenteritis (8). TlpA dose-dependently suppresses TLR/IL1 induced NF-κB activity and is thought to achieve this by competing with endogenous TIR domains to block downstream signaling (8). A similar mechanism is proposed for the *Staphylococcus aureus* TIR domain protein TirS which blocks TLR2-induced MAPK and NF-κB signaling (9).

Other Tcps, including TcpC from the uropathogenic *Escherichia coli* strain CFT073, TcpB/BtpA, and BtpB from *Brucella melitensis* which causes the chronic and debilitating zoonotic disease Brucellosis and ypTIR from the plague-causing *Yersinia pestis*, are all able to bind to MyD88 and prevent downstream signaling from TLRs (10–12). Additionally, TcpB was proposed to compete with the TIR-containing adaptor protein Mal/TIRAP to prevent TLR2- and TLR4-dependent signaling (13). TcpB downregulates Mal expression by targeting phosphorylated Mal for proteasomal degradation by a mechanism similar to the cellular SOCS1-mediated degradation of Mal (14).

## Bacterial Proteases

Several bacterial proteins can inhibit signaling by selectively cleaving signaling enzymes. *Bacillus anthracis* lethal factor (LF) is a protease that forms part of the anthrax toxin. LF specifically targets MAPK kinases (MKKs) by cleaving within the MAPK-docking domain (D-domain), which is required for binding to downstream substrates. LF-induced proteolysis disrupts or removes the D-domain to generate kinases that are unable to interact with downstream MAPKs, thereby blocking their phosphorylation and activation. Although originally described to block MKK1/2 (15), LF is capable of cleaving all MKKs except MKK5 (16), resulting in reduced kinase activity for ERK, p38, and JNK MAPK pathways.

Enteropathogenic and enterohemorrhagic *E. coli* (EPEC/EHEC) are closely related bacteria that cause severe food-borne gastroenteritis. Both use type III secretion systems (T3SS) to inject effector proteins into the host cell. One of these, NleD, is a zinc metalloprotease that inactivates JNK and p38 by cleaving between the dual phosphorylation sites within the kinase activation loop (17). Proteolysis as a strategy to dampen the immune response is not restricted to MAPKs as EPEC/EHEC proteases also target the NF-κB signaling pathway. NleC, another zinc protease, cleaves the p65 subunit of NF-κB at its amino-terminus to promote its proteasomal degradation (17–20) and has also been shown to target other NF-κB components including IκBα, p50, and c-Rel (19, 20). The NF-κB p65 subunit is also a target of proteolysis by the *Chlamydia* proteases CT441 (21, 22) and Chlamydial protease-like activity factor (CPAF) (23). CT441 inhibits NFκB activation by cleaving p65 at residue 351/2, which lies between the Rel-homologous domain and the transactivation domain (21).

## Bacterial Acetyltransferases

Some bacterial effectors modify host signaling proteins to inhibit their activity. Vibrio outer protein A (VopA) is an acetyltransferase expressed by *Vibrio parahaemolyticus* that inhibits signaling by all MAPKs through both O-acetylation (serine and threonine acetylation) and N-acetylation (lysine acetylation) of MAP2Ks (24, 25). VopA acetylates MKK6 on three residues (Ser207, Lys210, and Thr211) in the activation loop and on Lys172 in the catalytic loop. Phosphorylation of Ser207 and Thr211 by MAP3Ks is critical for MKK6 activation, and acetylation of these resides by VopA blocks MKK6 activity. Lys172 coordinates the γ-phosphate of ATP, and its N-acetylation disrupts ATP binding to prevent phosphorylation of downstream substrates. This dual approach of preventing kinase activation and locking the kinase in an inactive state makes VopA an extremely potent inhibitor of MAPK signaling.

*Salmonella* Typhimurium expresses the O-actetyltranferase AvrA that modifies the threonine residue in the activation loop of MKK4 to prevent JNK activation (26). An interaction between MKK7 and AvrA was observed in a yeast-two-hybrid screen (27) suggesting that it can act on both MKKs that activate JNK. Although overexpressed AvrA inhibits both p38 and JNK phosphorylation, only JNK phosphorylation is inhibited during *S*. Typhimurium infection and JNK target genes are upregulated in cells infected with ΔAvrA, lending support for AvrA being targeted to the JNK signaling pathway (27).

*Yersinia* species deliver *Yersinia* outer proteins (Yops) into the host cell via a Type III secretion system. The *Y. pestis*/*Yersinia pseudotuberculosis* effector YopJ (YopP in *Yersinia enterocolitica*) inhibits MAPK signaling by blocking the phosphorylation and activation of MAP2Ks (28). YopJ O-actetylates critical residues in the MAP2K activation loop, as described for MKK6 and MKK2 (29–31). In addition to targeting MAP2Ks, YopJ/YopP also inhibits the MAP3K TAK1 (31–34). YopJ O-acetylates Thr184 and Thr187 in the activation loop of TAK1, preventing Thr187 autophosphorylation and thereby blocking kinase activation. Conflicting reports exist regarding the effect of YopP on the formation of the TAK1-TAB2/3 complex, with one showing that YopP interferes with TAK1-TAB2 binding (32), while a second report demonstrated that it did not affect TAK1-TAB2/3 complex formation (33). YopP may also affect ubiquitination since overexpressed YopP blocks TRAF6-dependent polyubiquitination reactions, although the authors note that they were unable to reliably detect this effect on ubiquitination in *Yersinia*-infected cells (32). By acetylating both TAK1 and MKKs to prevent their activation, YopJ/P targets both the MAPK and NFκB signaling pathways. This dual targeting strategy is reinforced by the demonstration that YopJ also O-acetylates IKKα and IKKβ in the activation loop to inhibit their kinase activity and prevent activation of the NFκB pathway (28, 30).

Mitogen-activated protein kinases are inactivated by a number of different phosphatases of which the dual specificity phosphatase (DUSP) family members are key regulators of MAPK dephosphorylation in immunity. The Enhanced intracellular survival (Eis) protein of *Mycobacterium tuberculosis*, the causative agent of tuberculosis, targets the JNK pathway. Eis N-acetylates lysine 55 of the JNK-specific DUSP16, which is also known as MAPK phosphatase 7 or MKP7 (35). Lys55 lies within the substrate-docking domain of DUSP16, and its acetylation by Eis results in reduced JNK activity in cells. Similarly, DUSP1/MKP1 that has been acetylated on Lys57 by p300 reduces p38 activity (36). Acetylated DUSP1 binds more readily to p38, resulting in higher phosphatase activity and reduced p38 activity (36). Eis acetylation of DUSP16 is thought to act in a similar manner to reduce JNK activity.

## Bacterial Phosphothreonine Lyases

The OspF and SpvC proteins of *Shigella* and *S*. Typhimurium, respectively, target MAPK activation by specifically removing the phosphate group from phosphothreonine in the TxY activation loop (37, 38). Rather than acting as threonine-specific phosphatases, OspF and SpvC function as phosphothreonine lyases to irreversibly inactivate MAPKs via an eliminylation reaction whereby the threonine phosphate group is dephosphorylated by β-elimination to generate the unsaturated amino acid dehydrobutyrine (37, 39). The effect is irreversible as dehydrobutyrine lacks a hydroxyl group and cannot be phosphorylated. Although OspF and SpvC have activity against ERK, p38, and JNK (37, 38), OspF shows selectivity for ERK and p38 during *Shigella* infection (40, 41) and has actually been shown to potentiate JNK activity due to its phosphothreonine lyase activity on p38 disrupting a negative feedback loop between p38 and TAK1 (42).

## Bacterial Kinases

Some bacteria express their own kinases. For example *Shigella* OspG is a serine/threonine kinase that binds to ubiquitin and E2-ubiquitin conjugates in the SCF-βTrCP complex, dampening the host immune response by reducing IκBα degradation (43, 44). The interaction between OspG and ubiquitin activates its kinase activity, which is required for it to inhibit NFκB signaling. Binding of OspG to E2-ubiquitin conjugates also represses ubiquitin transfer to E3 ligases, as it has been shown to stabilize a UbcH5b-ubiquitin complex (45).

OspG shares significant sequence homology with the NleH family of proteins in *E. coli* and like OspG, NleH1, and NleH2 can inhibit IκBα ubiquitination to prevent its degradation (46). However, NleH1/2 is regulated differently to OspG, since their kinase activity is not induced by ubiquitin (44). Instead, NleH1 binds to a novel subunit of NFκB, ribosomal protein S3 (RPS3), antagonizing its function of guiding p65 to specific promoters and thereby reducing its transcriptional activity (47).

## Bacterial E3 Ligases

In addition to expressing kinases and phosphatases that can interfere with ubiquitin-dependent signaling, bacteria also use their own E3 ligases and deubiquitinase. IpaH proteins belong to the Novel E3 Ligase (NEL) family of ubiquitin E3 ligases of which *Shigella* IpaH9.8 and *Salmonella* SspH1 were the first described members (48). Although a large number of bacterial E3 ligases have been identified (49) many of their ubiquitination targets are unknown. Using the yeast Saccharomyces cerevisiae as a model system, it was demonstrated that IpaH9.8 acts as an E3 ligase for the MAPKK Ste7 (48), and that in human cells IpaH9.8 mediates lysine-27 polyubiquitination of NEMO/IKKγ resulting in the degradation of both proteins (50). Other IpaH family members also possess E3 ligase activity, with *Shigella* IpaH4.5 ubiquitinating p65 to block NF-κB transcription (51) and IpaH0722 targeting TRAF2 for ubiquitin-dependent degradation to inhibit PKC-induced NF-κB activity (52). Interestingly reports are now emerging of bacterial E3 ligases targeting other aspects of immune signaling. For example, *Shigella* IpaH7.8 activates the inflammasome, resulting in cell death and enhanced bacterial replication (53).

## Bacterial Deubiquitinases (DUBs)

Deubiquitinases (DUBs) are proteases that remove ubiquitin from proteins. Both the AvrA and SseL proteins of *S*. Typhimurium and the ChlsDub1 protein of *Chlamydia trachomatis* possess DUB activity that inhibits K48-linked ubiquitination and degradation of IκBα, thus blocking NFκB activation (54–57). In addition to its DUB activity, ChlsDub1 has also been shown to have deNEDDylating activity (59) that may contribute to suppressing IκBα degradation by antagonizing conjugation of the ubiquitin-like NEDD8 protein to the SCF-βTrCP complex, although this has not been formally demonstrated.

*Burkholderia (Pseudomonas) pseudomallei*, which causes melioidosis, expresses the effector protein TssM, which possesses DUB activity against both K63 and K48-linked polyubiquitin (58, 60). TssM overexpression causes reduced K48-linked polyubiquitination of IκBα and reduced K63-linked polyubiquitination of TRAF6 to block NFκB-induced transcription (60).

## Other Bacterial Effectors

In addition to the above proteins, further bacterial effectors that block MAPK and NFκB activity via different mechanisms have started to emerge. *M. tuberculosis* tyrosine phosphatase PtpA dephosphorylates JNK and p38 to dampen cytokine expression (61). PtpA uses a novel mechanism whereby its phosphatase activity is stimulated by binding to ubiquitin via a novel ubiquitin-interacting motif-like (UIML) region (61). Mtb PtpA also suppresses NFκB activation by competitively binding to the Npl4 zinc finger domain (NZF) of TAB3, blocking its ability to bind to ubiquitin chains and thereby reducing Tak1 activity.

The *Shigella flexneri* type III effector OspI blocks TRAF6 mediated signaling by selectively deamidating a glutamine residue to glutamic acid in the E2 enzyme Ubc13 (62). The deamidation of Glu100 prevents Ubc13 from binding to TRAF6, inhibiting its E3 ligase activity and thereby blocking downstream signaling.

The EPEC NleE protein also uses a unique mechanism to inhibit bacterial-induced signaling. NleE is an S-adenosyl-l-methionine (SAM)-dependent cysteine methyltransferase that targets the Npl4 zinc finger (NZF) domains in TAB2/3 (63). Modification of cysteine residues in the zinc finger domains of TAB2/3 abolishes their ability to bind to Lys63-linked polyubiquitin chains and therefore blocks downstream TAK1 activation, consistent with the observed inhibition of IκBα phosphorylation and NFκB signaling in the presence of NleE (64, 65). NleE proteins from other pathogens, such as *S. flexneri* protein OspZ, were shown to be functionally interchangeable with NleE in blocking NFκB signaling and may also act as a cysteine methyltransferases (65).

## Summary and Future Perspectives

Bacterial pathogens have evolved diverse and elegant ways to block MAPK and NF-κB signaling downstream of TLR activation, enabling them to evade detection by the immune system and promote infection. Many bacteria employ strategies to simultaneously target a number of proteins within these, as well as other host pathways, to increase their chances of overcoming the immune response. Future discoveries in understanding how and why pathogens target particular proteins will not only demonstrate their importance in immunity, but will also help our understanding of how bacteria activate intracellular signaling pathways, and have the potential to identify new targets for the treatment or prevention of infection.

## Author Contributions

Both VM and SA wrote the article.

## Conflict of Interest Statement

The authors declare that the research was conducted in the absence of any commercial or financial relationships that could be construed as a potential conflict of interest.
